# Oral health status and quality of life in female patients receiving low dose bone-modifying agents for cancer treatment-induced bone loss: a single-center exploratory study

**DOI:** 10.3389/froh.2025.1683722

**Published:** 2025-10-31

**Authors:** Rodolfo Mauceri, Martina Coppini, Sara Maria Marchese, Nicola Mauceri, Rita Coniglio, Marco Nisi, Maria Elena Mauceri, Vittorio Fusco, Maria Rosaria Valerio, Giuseppina Campisi

**Affiliations:** 1 Department of Precision Medicine in Medical, Surgical and Critical Care, University of Palermo, Palermo, Italy; 2 Unit of Oral Medicine and Dentistry for Frail Patients, Department of Rehabilitation, Fragility, and Continuity of Care, Regional Center for Research and Care of MRONJ, University Hospital Palermo, Palermo, Italy; 3 Department of Biomedical and Dental Sciences and Morphofunctional Imaging, University of Messina, Messina, Italy; 4 Department of Biomedicine, Neuroscience and Advanced Diagnostics (Bi.N.D), University of Palermo, Palermo, Italy; 5 Department of Surgical Pathology, Medicine, Molecular and Critical Area, University of Pisa, Pisa, Italy; 6 Department of Biological, Chemical and Pharmaceutical Sciences and Technologies (STEBICEF), University of Palermo, Palermo, Italy; 7 Oncology Unit, Department of Medicine and Translational Medicine Unit, DAIRI-Department of Integration, Research and Innovation, “SS Antonio e Biagio e C. Arrigo” Hospital, Alessandria, Italy; 8 Medical Oncology Unit, University Hospital “Policlinico P. Giaccone,” University of Palermo, Palermo, Italy

**Keywords:** osteonecrosis of the jaw, ONJ, MRONJ, breast cancer, cancer treatment-induced bone loss, CTIBL, bone modifying agents, PSR

## Abstract

**Introduction:**

Breast cancer patients, undergoing low-dose bone-modifying agent (LD-BMA) therapy for cancer treatment-induced bone loss (CTIBL), represent an emerging category at risk of Medication-Related (MRONJ) low (<1%) but not absent. However, data on their oral health status and quality of life related are currently limited. This single-center exploratory study aimed to assess oral health conditions, periodontal status, and oral health-related quality of life in non-metastatic breast cancer patients under LD-BMA therapy for CTIBL.

**Materials and methods:**

Forty patients were consecutively and unselectively enrolled and divided into two groups based on the duration of LD-BMA therapy (<3 years vs. ≥3 years). Oral examination by decayed-missing-filled teeth index (DMFT) and Periodontal Screening and Recording (PSR) was performed, and the OHIP-14 questionnaire was administered to assess their oral health-related quality of life.

**Results:**

No statistically significant differences were observed between the two groups in PSR, DMFT, or OHIP-14 scores. PSR scores indicating moderate-to-severe periodontal involvement (3–4) were reported in 73.3% of patients treated for <3 years and 70% of those treated ≥3 years. Mean DMFT values were 9.7 and 12.0, respectively. Although patients treated for ≥3 years reported higher OHIP-14 scores, this trend did not reach statistical significance. No cases of MRONJ were observed in the study groups.

**Conclusions:**

Patients affected by breast cancer receiving LD-BMA therapy for CTIBL and recruited in a preventive program appear to have a very low risk of MRONJ. Despite comparable clinical findings across treatment durations, longer LD-BMA therapy may be associated with a perceived reduction in oral well-being, possibly related to systemic and psychosocial burdens. These findings, with the limitation of a small sample size, support the implementation of individualized, risk-based dental and psychological preventive strategies, and reinforce the relevance of long-term dental surveillance in this under-explored population.

## 1 Introduction

Medication-related osteonecrosis of the jaw (MRONJ) has been defined as an “adverse drug reaction described as the progressive destruction and death of bone that affects the mandible and maxilla of patients exposed to the treatment with medications known to increase the risk of the disease, in the absence of a previous radiation treatment” (1).

Currently, four categories of patients are reported at risk of MRONJ: (1) cancer patients with bone metastases or with multiple myeloma, and (2) patients affected by Giant Cell Tumour of Bone, both usually receiving high doses of BMA (HD-BMA); (3) osteoporotic patients, and (4) patients affected by breast cancer or prostate cancer without bone metastases, both treated with low doses of BMA(LD-BMA) (1, 2).

The fourth group is an emerging category of patients at risk of MRONJ under hormonal therapy, who mostly receive LD-BMAs at the same dosage as osteoporotic patients, to prevent Cancer Treatment-Induced Bone Loss (CTIBL) (3–5). Theoretically, this group possesses a low risk of developing MRONJ as they take LD-BMAs (<1%), but, simultaneously, they have systemic risk factors similar to those in cancer patients. Additionally, they remain at constant risk of developing bone metastases, which may necessitate a switch to HD-BMA therapy, drastically increasing their MRONJ risk (5, 6).

A recent study reported that, among patients affected by breast cancer with bone metastases receiving HD-BMA therapy, the incidence of MRONJ ranged from 2.8% and 16.3%, according to the specific BMA used, being lowest with bisphosphonates (BP) alone and highest in patients treated with denosumab (DNB) or sequentially with BP followed by denosumab (7).

To date, several MRONJ recommendations have agreed on the importance of primary dental prevention measures to reduce the MRONJ risk (1, 8–10). Oral health specialists should control and modify the local risk factors of MRONJ (e.g., dental, periodontal, periapical, and peri-implant infection) since the identification, management, and, when possible, elimination of local risk factors has so far demonstrated the greatest success in reducing the incidence of MRONJ (9–16).

According to the majority of clinical recommendations, a dental evaluation is not explicitly mandatory for patients before starting LD-BMAs; however, it is also recommended within the first six months of treatment (1, 5, 8).

For this reason, initial and periodic oral health status assessment, including clinical and radiological examinations, is crucial to identify and treat common oral diseases potentially related to MRONJ onset (1).

Very useful for this goal are the decayed-missing-filled teeth index (DMFT) and periodontal screening and recording (PSR), able to assess the dental and periodontal health status through a rapid and effective procedure, which avoids unnecessary waste of human and economic resources (17, 18).

Finally, previous studies identified that cancer patients have a poorer oral health-related quality of life (OHRQOL), which may be attributable to both the underlying disease and adverse effects associated with cancer treatment (19). A better OHRQOL is associated with prolonged survival and reduced hospitalization risk (20). Therefore, maintaining a good quality of oral health-related quality of life should be considered one of the primary goals in the management of cancer patients. Nevertheless, only a few studies have explored oral health-related quality of life in cancer patients, and none have specifically addressed patients undergoing LD-BMA therapy for CTIBL prevention (19, 21, 22). To explore the impact of cancer disease and MRONJ risk on quality of life, the short-form Oral Health Impact Profile (OHIP-14) is considered a useful and efficient test (23).

The present study aims to evaluate for the first time in the literature both dental and periodontal health status (by DMFT and PSR) and oral health-related quality of life (by OHIP-14) in breast cancer patients undergoing LD-BMA therapy for CTIBL. The findings of this exploratory study may contribute to profiling primary prevention programs both before starting and during LD-BMA therapy in this category of cancer patients.

## 2 Materials and methods

### 2.1 Study design

The present single-center cross-sectional exploratory study included consecutive and unselected non-metastatic breast cancer patients receiving LD-BMAs for CTIBL prevention. Patients' data were retrospectively collected from the Oral Medicine Unit “Valerio Margiotta” of the University Hospital “Paolo Giaccone” of Palermo (Italy), between December 2023 and January 2025. The study was conducted according to the ethical guidelines of the Declaration of Helsinki (1964) and its later amendments or comparable ethical standards, and it was approved by the Ethical Committee of “Paolo Giaccone” University Hospital of Palermo (#1/2022). Written informed consent was obtained from all participants involved in the study. The study was conducted following the STROBE Statement for Observational Cohort Studies (24).

### 2.2 Eligibility criteria

Patients affected by breast cancer without bone metastases receiving LD-BMAs for CTIBL who underwent the first clinical dental examination at the Oral Medicine Unit of the University Hospital “Paolo Giaccone” of Palermo (Italy) between December 2023 and January 2025 were assessed.

The inclusion criteria in the study cohort were the following:

- age ≥ 18 years;
- women affected by breast cancer and treated with adjuvant endocrine therapy;
- current treatment with low-dose BMAs for CTIBL prevention.

The exclusion criteria were a history of high-dose BMAs for bone metastases, concurrent use of anti-angiogenic agents or other drugs at risk of MRONJ onset, and exposure to radiant therapy of the head and neck.

### 2.3 Protocol study

Patients assuming LD-BMAs underwent dental and radiological examinations at the Oral Medicine Unit of the “Paolo Giaccone” University Hospital in Palermo (Italy). A personalized follow-up plan was established as part of a preventive strategy following institutional protocols and regulatory guidelines adopted by the same hospital (25, 26). In general, this consisted of periodic visits every six months; however, in the presence of local risk factors, including periodontitis, follow-up appointments and periodontal therapy were scheduled more frequently. Specifically, patients presenting PSR scores of 3 or 4 underwent evaluation of periodontal damage, periodontal debridement, and subsequent re-evaluation (26).

Patients' data were retrospectively collected through a review of patient charts, and dental and periodontal health status were assessed and recorded through DMFT and PSR indices.

The number of Decayed Teeth (D), Missed Teeth (M), and Filled Teeth (F) was recorded in the DMFT form (27). Third molars were excluded, thus being the maximum possible score of 28 points.

The DMFT index has been extensively applied in oral health research, including studies in cancer patients, because it is simple, quick, and standardized, allowing for reliable assessment and comparison of patients' dental health status (28–31).

The PSR index is a rapid, standardized, and widely used screening tool in epidemiological and clinical studies. It is considered quick, reliable, reproducible, and cost-effective, while also facilitating record keeping, risk management, and patient education (17, 32, 33).

Periodontal examination was performed with a manual periodontal probe of the World Health Organization. The probe has a rounded tip of 0.5 mm in diameter and a colourful area that extends from 3.5 mm to 5.5 mm. The correct application of the PSR consists of carrying out a complete objective examination of all the surfaces of all dental elements by dividing the mouth into six sextants. Each sextant was evaluated according to the following PSR criteria scale (34):

– Code 0: health. The coloured portion of the probe remains completely visible even at the maximum probing point of the sextant. No plaque, tartar or protruding restoration margins are detected. No bleeding is detected on probing.
– Code 1: gingivitis. Like code 0 but with bleeding on probing.
– Code 2: like code 0 but with calculus.
– Code 3: chronic periodontitis with early or moderate attachment loss. The coloured portion of the probe remains only partially visible at the point of maximum probing of the sextant. This indicates the presence of a pocket between 3.5 and 5.5 mm deep.
– Code 4: chronic periodontitis with moderate attachment loss or a form of aggressive periodontitis. The coloured portion of the probe disappears completely at the point of maximum probing of the sextant. This indicates the presence of a pocket greater than 5.5 mm in depth.

The Oral Health Impact Profile (OHIP) is the most used tool to assess individuals' perception of the impact of oral disorders on their quality of life, and it has been particularly applied in studies involving cancer patients (35–39). In the present study, to assess the OHRQOL, the short-form of OHIP-14 was administered to all patients. This tool consists of 14 items organized into seven categories (i.e., functional limitations, physical pain, psychological discomfort, physical disability, social disability, and handicap) (40). Responses are measured in a dichotomous manner (yes/no) (41). The sum of such ratings from the 14 questions generates a total score that could range from 0 to 14, where higher scores indicate a greater impact on quality of life. Specifically, scores from 1 to 4 indicate a minimal impact, scores from 5 to 9 reflect a moderate impact, and scores from 10 to 14 represent a high impact on oral health-related quality of life.

### 2.4 Study variables

The following data were recorded in all recruited cases: demographic data; BMA therapy (i.e., type, dose); duration of BMA therapy at the moment of the visit; oral health status (i.e., DMFT); periodontal status (i.e., PSR); oral health-related quality of life (i.e., OHIP-14), clinical variables associated with MRONJ-risk (e.g., smoking habits, comorbidities such as diabetes, concomitant corticosteroid treatment); potential oral triggers associated with MRONJ (e.g., periodontitis, dentures, tooth extraction, etc.) and eventual onset of MRONJ (according to SIPMO-SICMF clinical-radiological staging system) (1).

The DMFT index, PSR, and OHIP-14 questionnaire were selected as they remain among the most widely used and validated measures for oral health status and oral health-related quality of life, as confirmed by recent studies (42–44).

Based on the literature data, patients were divided into two groups according to the duration of LD-BMA therapy (45–47):

– group A: <3 years of LD-BMA treatment
– group B: ≥3 years of LD-BMA treatment

### 2.5 Statistical analyses

Continuous or ordinal variables were compared between the two groups using the nonparametric Mann–Whitney U test, given the small sample size and the ordinal nature of several variables (e.g., DMFT, PSR, OHIP-14). Categorical variables were analyzed using Fisher's exact test. Correlations between continuous or ordinal variables were evaluated using Spearman's rank correlation coefficient. Statistical significance was set at *p* < 0.05.

## 3 Results

A total of 40 breast cancer patients under LD-BMA therapy for the prevention of CTIBL were included in the study.

The mean age of patients was 63 ± SD 9.9 years, with a median of 63 years. (range 45–79).

Regarding BMA therapy, 17 patients received alendronate, 5 clodronate, 4 risedronate, and 14 denosumab (biannual dose of 60 mg).

Based on LD-BMA therapy duration, the included patients were analysed and divided into two groups (group A <3 years vs. group B ≥ 3 years). In both groups, no cases of MRONJ were observed at the moment of the dental visit.

The main features of the patients are reported in Table 1.

**Table 1:** Study population features.

N. 40 Breast cancer patients under LD-BMA therapy for CTIBL (%)		
Features	Duration of BMA therapy <3 years (n. 30)	Duration of BMA therapy >3 years (n. 10)
Median age (years)	63	66.5
Mean age ± SD (years)	62.2 ± 8.4	65.4 ± 13.1
Range	45–78	46–79
LD-BMA therapy		
Alendronate^a^	12 (40%)	5 (50%)
Clodronate^a^	5 (16.67%)	–
Risedronate^a^	2 (6.67%)	2 (20%)
Denosumab^a^	11 (36.67%)	3 (30%)
Mean time of LD-BMA therapy ± SD (months)		
Alendronate	18.6 ± 6.2	46.2 ± 12.5
Clodronate	21.2 ± 4.2	–
Risedronate	5.5 ± 1.5	51 ± 3
Denosumab	12.2 ± 7.4	86.6 ± 24.9
Median time of LD-BMA therapy and range (months)		
Alendronate	19 (10–30)	36 (36–63)
Clodronate	24 (13–24)	–
Risedronate	5.5 (4–7)	51 (48–54)
Denosumab	12 (4–24)	84 (60–120)
Comorbidities		
Hypertension	10 (33.33%)	7 (70%)
Diabetes	3 (10%)	1 (10%)
Arthrosis	1 (3.33%)	1 (10%)
HCV-related hepatopathy	1 (3.33%)	1 (10%)
Smokers	5 (16.66%)	1 (10%)
PSR		
1–2	8 (26.67%)	3 (30%)
3–4	22 (73.33%)	7 (70%)
Decayed Teeth (D) for patient		
No DT	13 (43.33%)	4 (40%)
1	6 (20%)	4 (40%)
2	8 (26.66%)	2 (20%)
3	3 (10%)	–
Missed Teeth (M) for patient		
No MT	4 (13.33%)	1 (10%)
1–3	7 (23.33%)	4 (40%)
4–6	11 (36.66%)	2 (20%)
7–9	4 (13.33%)	1 (10%)
≥10	4 (13.33%)	2 (20%)
Filled Teeth (F) for patient		
No FT	4 (13.33%)	1 (10%)
1–3	14 (46.66%)	2 (20%)
4–6	6 (20%)	2 (20%)
7–9	3 (10%)	1 (10%)
≥10	3 (10%)	2 (20%)
DMFT		
0	3 (10%)	–
1–5	3 (10%)	2 (20%)
6–10	12 (40%)	2 (20%)
11–15	7 (23.33%)	3 (30%)
16–20	4 (13.33%)	2 (20%)
≥21	1 (3.03%)	1 (10%)
OHIP-14		
Minimal impact	22 (73.33%)	4 (40%)
Moderate impact	6 (20%)	5 (50%)
High impact	2 (6.67%)	1 (10%)
Tooth extraction	12 (40%)	7 (70%)
Before BMA therapy	8/30 (26.66%)	0
During BMA therapy	4/30 (13.33%)	7/10 (70%)
<6 months	2/30 (6.66%)	0
6–12 months	2/30 (6.66%)	2/10 (20%)
12–24 months	0	2/10 (20%)
≥36 months	0	3/10 (30%)
Dental implants	6 (20%)	0
Dentures	9 (30%)	2 (20%)
MRONJ onset	0	0

a Dosing regimens were as follows: Alendronate 70 mg orally once weekly; Clodronate 100 mg intramuscularly once weekly or 200 mg every two weeks; Risedronate 35 mg orally once weekly; Denosumab 60 mg subcutaneously every six months.

No statistically significant differences were observed with respect to age, smoking habits, or comorbidities.

Although 26.7% of patients in Group A (8 out of 30) had undergone tooth extractions before LD-BMA therapy compared to none in Group B, the difference was not statistically significant (Fisher's exact test, *p* = 0.165).

During therapy, a significantly higher proportion of patients in group B (7 out of 10) underwent an intercurrent tooth extraction (2 in the first year, 2 in the second year, and 3 afterwards), compared to those in Group A (4 out of 30, all during the first year) (*p* = 0.0015):

Among group A patients, 6 had dental implants (30%) and 9 were wearing removable dentures (30%); in group B, 2 patients (20%) wore dentures.

Regarding decayed teeth (D), 43.3% of patients in group A had no decayed teeth, compared to 40% in group B. Mild decay (1–2 teeth) was slightly common in group A (60%) than in group B (46.7%). Severe decay (≥3 teeth) was observed only in few patients in group A (10%).

For missing teeth (M), in group A, the highest proportion of patients belonged to the group with 4–6 missing teeth (36.7%), while in group B, the highest proportion of patients belonged to the category with 1–3 missing teeth (40%).

Regarding filled teeth (F), the most represented category in group A was 1–3 filled teeth (46.7%). In contrast, group B showed a more heterogeneous distribution, with 20% of patients presenting with ≥10 restorations.

DMFT scores revealed that most patients in group A clustered within the 6–15 range (63.3%), whereas patients in group B showed a more heterogeneous distribution across the full range of scores, from 1 to over 21.

**Figure 1:**
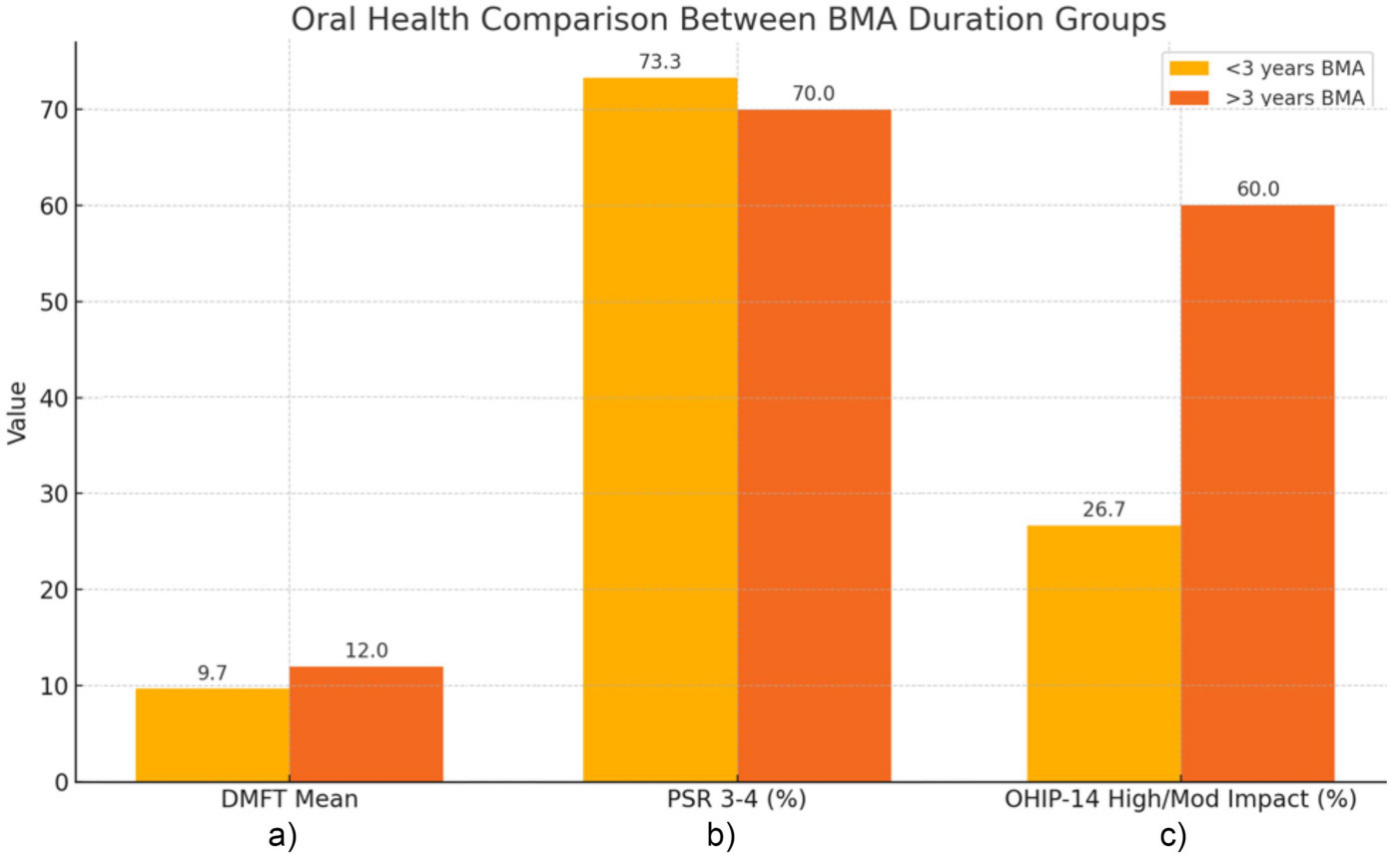
Comparison of oral health outcomes between groups according to BMA therapy duration. In detail: **(a)** Mean DMFT in Group **A** and Group **B**; **(b)** Prevalence of PSR scores 3–4 in Group **A** and Group **B**; **(c)** Proportion of patients reporting moderate or high OHIP-14 impact in Group A and Group B.

The comparison of oral health outcomes between group A and B is reported in Figure 1. The mean estimated DMFT score was 9.7 among patients in group A and 12.0 among patients in group B, with no statistically significant difference between the groups (*p* = 0.342). Moreover, Spearman's correlation showed a weak positive value between LD-BMA therapy duration and DMFT scores (*ρ* = 0.16).

Regarding periodontal status, PSR scores of 1–2 were recorded in 26.7% and 30% of patients in groups A and B, respectively. PSR scores of 3–4, indicative of moderate-to-severe periodontal involvement, were observed in 73.3% of patients in group A and 70% of those in group B, showing a comparable distribution between the two cohorts. No significant correlation was observed between LD-BMA therapy duration and PSR scores (*ρ* = –0.06, *p* = 0.72).

Finally, to evaluate the oral health-related quality of life, the OHIP-14 questionnaire was administered. Among patients of group A, the impact on quality of life was reported as minimal in 22 patients (73.33%), moderate in 6 (20%), and high in 2 (6.67%). In group B, 4 patients reported a minimal impact (40%), 5 a moderate impact (50%), and 1 a high impact (10%).

Although patients in group A more frequently reported a minimal impact on oral health-related quality of life (73.3% vs. 40%), the difference in OHIP-14 score distribution between the two groups was not statistically significant (*p* = 0.146). However, the higher proportion of moderate and high impact in group B may suggest a trend toward reduced perceived oral well-being attributable to the longer treatment duration and possible consequences or risk of MRONJ, especially if switching to HD-BMA.

A borderline moderate positive correlation was found between LD-BMA therapy duration and OHIP-14 impact scores (*ρ* = 0.29), suggesting a possible trend toward reduced perceived oral quality of life with longer treatment.

## 4 Discussion

The present study aims to assess dental and periodontal health status and quality of life in patients under LD-BMA therapy for CTIBL.

The oral clinical scenario of this patient category has been poorly investigated in the literature, as well as their MRONJ risk (6).

This study explores, for the first time to the best of our knowledge, both dental and periodontal health status (DMFT and PSR scores) and oral-health related quality of life (OHIP-14 questionnaire) in this specific patient population.

Breast cancer is the most commonly diagnosed malignancy worldwide and represents the leading cause of cancer-related death among women. According to GLOBOCAN 2022 estimates, breast cancer accounted for approximately 11.6% of all new cancer cases globally, making it the second most common cancer after lung cancer. In terms of mortality, breast cancer was responsible for 6.9% of all cancer deaths. Specifically, there were an estimated 2.3 million new cases and 670,000 deaths from breast cancer worldwide in 2022 (48).

In patients operated for breast cancer (at risk of tumor recurrence and of metastatic disease) and undergoing “adjuvant” therapies (sometimes including LD-BMAs), treatments could potentially compromise oral function and quality of life. Moreover, patients receiving LD-BMAs for prevention of CTIBL (as well as osteoporotic patients) are considered a group at relatively low risk of MRONJ development (in comparison to the high risk of metastatic cancer patients treated with HD-BMAs) (49): so they are a “borderline” population, since they share common features of both cancer patients and osteoporotic patients (6).

Moreover, it is estimated that 15%–25% of all breast cancer patients will eventually develop bone metastases during their life, with possible need of switching from LD to HD-BMA therapy, consequently increasing the risk of MRONJ onset (50, 51).

In general, the literature shows that the risk of developing MRONJ in individuals receiving LD-BMA therapy is influenced by both the length of exposure and the cumulative dose (1). Notably, the risk typically emerges between two and five years after therapy initiation, depending on the specific agent used. The authors of the present study decided to consider the main threshold of the LD-BMA assumption as a time ≥ 3 years (45–47).

In the present study, 40 breast cancer patients undergoing LD-BMA therapy for CTIBL were analyzed, assessing dental and periodontal health status and OHRQOL, distributed in 2 groups (<3yrs of LD-BMA vs. ≥3 years of LD-BMA).

According to the Mann–Whitney and Fisher's exact tests, no statistically significant differences were found between the two groups in PSR, DMFT, or OHIP-14 scores. Interestingly, the clinical conditions observed in our cohort appeared more favourable than those reported in previous studies on healthy individuals (36, 37). This finding may be explained, at least in part, by an unintentional selection bias favouring patients who were more closely monitored by their oncologists or who voluntarily sought dental care beyond the minimum standard follow-up.

In the present study, the mean estimated DMFT score was 9.7 and 12.0 among patients of groups A and B, respectively, and in a study conducted by Skaleric et al., the mean DMFT value among healthy individuals aged 45 to 95 years was 19.3 (52); similarly, Pawinska et al. reported mean DMFT scores of 21.9 ± 5.1 in patients aged 55–64 and 23.8 ± 5.2 in those aged 65–74 (53).

Similarly to dental status, no statistically significant differences in periodontal condition were observed between the two groups, as most patients in both groups presented with PSR scores of 3–4. These values are consistent with those reported in previous studies on healthy adult individuals (54–56).

According to a meta-analysis performed by Trindade et al., the prevalence of periodontitis among healthy individuals aged 65 years and older reaches 79% (57). Consistently, a recent cross-sectional study assessing periodontal health status in older adults reported that the prevalence of moderate or severe periodontitis increases with age, reaching 94.9% among individuals aged 60 to 69 years (58).

In terms of quality of life, although no statistically significant differences were found between the two study groups, a higher proportion of patients under BMA therapy for more than 3 years reported moderate to high impact on oral health-related quality of life compared to patients under BMA therapy for less time (60% vs. 26.7%), suggesting a possible trend toward reduced perceived oral well-being with longer BMA treatment duration, potentially also influenced by increased patient awareness of their cancer disease progression and concern related.

Tooth extractions were required in 13.3% of patients undergoing BMA therapy for less than three years, compared to 70% of group B. This difference may reflect the fact that, over time, compromised teeth are more likely to be extracted electively as part of preventative management strategies aimed at reducing the risk of MRONJ, rather than simply a worsening oral health over time. These findings highlight the importance of prevention in LD-BMA users, especially those who have been using it for more than three years.

Additionally, it is plausible that the greater awareness of MRONJ risk among patients undergoing LD-BMA therapy for more than three years contributes to their increased attention to oral health. This may result in better adherence to regular dental check-ups and improved compliance with oral hygiene practices, which could, in turn, partially explain the better PSR values observed in this group compared to Group A (59).

The concern of patients in group B appears to be justified, as cases of MRONJ have been reported both in patients continuing LD-BMA therapy for more than 3 years due to BMA cumulative dose or BMA therapy prolongation, and in those who later switched to HD-BMA regimens due to the onset of bone metastases (6, 14, 60).

In our previous multicentric study, 15 breast cancer patients under LD-BMA therapy for CTIBL developed MRONJ after a mean duration of 35.7 months of LD-BMA therapy (±26.3 months, median 24) (6). None of these patients had undergone dental visits before starting LD-BMA therapy, and when they presented to our attention, they already exhibited clear clinical signs and symptoms of established MRONJ. These cases may be explained by the absence of a preventive MRONJ program and by the presence of significant local risk factors (e.g., periodontal disease, peri-implantitis) (1, 61).

Accordingly, another study reported the MRONJ onset in 2 patients affected by breast cancer after switching from LD to HD-BMA due to bone metastases (60).

Moreover, a recent longitudinal Swedish study investigating the incidence of MRONJ in breast cancer patients receiving LD-BMA and HD-BMA therapy reported no cases of MRONJ among the 119 patients treated exclusively with biannual zoledronic acid; however, the follow-up period was relatively short, with a median treatment duration of less than two years. In contrast, one out of nine patients who were shifted from LD to HD BMA following the onset of bone metastases developed MRONJ (14).

These findings highlight the importance of long-term monitoring and tailored preventive strategies in this patient population due to their peculiar clinical profile based on:

– cumulative dose or prolonged therapy in patients receiving LD-BMA therapy;
– increased MRONJ risk in patients switching to HD-BMA due to the bone metastases.

Given their dynamic risk of MRONJ onset, a multidisciplinary approach is crucial for effective prevention in this group (2, 6, 14).

In detail, the primary aim of MRONJ prevention, both before and during BMA therapy, is to maintain or re-establish oral and periodontal health through various procedures, including regular dental check-ups and professional hygiene, the implementation of minimally invasive procedures to control local risk factors, and the timely execution of invasive treatments, such as extractions, when teeth are deemed non-restorable (26, 62, 63). Moreover, a fundamental component of an efficient primary prevention is the patient's awareness. Unfortunately, it was reported that patients receiving BMA therapy are often unaware of the MRONJ risk and appropriate preventive strategies (2, 64). Patients, both under LD-BMA and HD-BMA therapies, should be informed about the risk of MRONJ and educated on its potential clinical signs and symptoms to facilitate early recognition and timely intervention. The dental team, including the dentist and dental hygienist, plays a crucial role in promoting awareness of the importance of oral health and the potential for oral adverse drug reactions, such as MRONJ (65).

The present study has several limitations, primarily related to the small sample size, particularly in group B, which may reduce the statistical power and limit the ability to detect subtle differences. Given the small sample size, nonparametric tests (Mann–Whitney and Fisher's exact) were applied to ensure robust comparisons between groups. Nonetheless, the exploratory nature and limited statistical power of the study restrict generalization of inferential results.

The limited number of participants is mainly due to the monocentric nature of the study and the fact that it involves an emerging and relatively under-explored patient category. It is conceivable that expanding the sample size in future multi-center investigations may lead to statistically significant results and more robust conclusions. Furthermore, the follow-up period is relatively short, not exceeding 4 years from the start of LD-BMA therapy.

The OHIP-14 was administered with dichotomous answers (yes/no) rather than with the standard Likert scale. This approach, while potentially less sensitive, was chosen to improve feasibility and reduce response bias, as patients often tend to select intermediate values on Likert scales to avoid taking a clear position (66, 67).

Lastly, the absence of MRONJ cases in this study must be carefully interpreted, as it may reflect not only the preventive strategies adopted but also the specific characteristics of a specialized university clinic setting and the limited sample size, which could limit the generalizability of the findings.

Being a monocentric and exploratory study conducted within a specialized preventive program, the findings may not be representative of the broader breast cancer population under LD-BMAs, contributing to the unexpectedly favourable oral health indices compared with general population data.

Longitudinal studies with larger cohorts are needed to confirm these findings and monitor oral health status over time in patients receiving long-term LD-BMA therapy. Furthermore, comparing different BMA agents (e.g., bisphosphonates *vs*. denosumab) in terms of their long-term oral effects could inform personalized treatment planning and follow-up protocols. Integrating psychological assessment with OHIP-14 could also help elucidate the impact of cancer therapy on patients' perceived oral health and quality of life.

## 5 Conclusion

The present study confirms that patients affected by breast cancer under LD-BMA show a very low risk of MRONJ onset and that they have dental and periodontal indices similar to those of the general population. The only difference observed between the two study subgroups was that patients undergoing LD-BMA therapy for three years or more reported a lower oral health-related quality of life compared to those treated for a shorter duration. Therefore, preventive strategies are strongly recommended in all cases, both before and during BMA therapy. However, in patients for whom an LD-BMA treatment is planned for three years or more, dental and psychological preventive measures should be even more emphasized to improve their quality of life and reduce their MRONJ risk.

## Data Availability

The raw data supporting the conclusions of this article will be made available by the authors, without undue reservation.
